# Diagnostic yield of quantitative endotracheal aspirates in patients with severe nursing home-acquired pneumonia

**DOI:** 10.1186/cc5917

**Published:** 2007-05-17

**Authors:** Ali A El Solh, Morohunfolu E Akinnusi, Lilibeth A Pineda, Corey R Mankowski

**Affiliations:** 1 Western New York Respiratory Research Center, Division of Pulmonary, Critical Care, and Sleep Medicine, Department of Medicine, State University of New York at Buffalo School of Medicine and Biomedical Sciences, Grider Street Buffalo, New York 14215, USA

## Abstract

**Introduction:**

Diagnostic strategies based on tracheal aspirates in patients with severe nursing home-acquired pneumonia have not previously been evaluated. The objectives of the study were to investigate, in patients with severe nursing home-acquired pneumonia, the diagnostic value of quantitative endotracheal aspirate (QEA) cultures using increasing interpretative cutoff points, as compared with bronchoalveolar lavage (BAL) and protected specimen brush (PSB) quantitative cultures.

**Methods:**

Seventy-five nursing home patients requiring mechanical ventilation for suspected pneumonia were studied. Endotracheal aspirate, PSB, and BAL samples were obtained consecutively. The diagnostic yield of QEA at thresholds raging from 10^3 ^to 10^7 ^colony-forming units (cfu)/ml was assessed by calculating sensitivities, specificities, and accuracy rates. A receiver operator characteristic curve for the series of cutoff points was constructed.

**Results:**

Forty-nine patients were diagnosed with pneumonia either by BAL (≤ 10^4 ^cfu/ml) or PSB (≤ 10^3 ^cfu/ml). Diagnostic accuracy of QEA was most favorable at 10^4 ^cfu/ml. At this threshold, endotracheal aspirates coincided with both BAL and PSB in 30 cases, whereas partial agreement was observed in 14 cases. This resulted in sensitivity and specificity of 90% (95% confidence interval 78% to 97%) and 77% (95% confidence interval 56% to 91%), respectively. QEA findings correlated significantly with both PSB and BAL quantitative cultures (*r *= 0.71 [*P *< 0.001] and *r *= 0.77 [*P *< 0.001], respectively).

**Conclusion:**

QEA may be used as a diagnostic tool to determine the presence of pneumonia in ventilated patients admitted from nursing homes when bronchoscopic procedures are not feasible or available.

## Introduction

Nursing home-acquired pneumonia (NHAP) is the leading cause of death among long-term care patients and is one of the most common causes of transfer to hospital, where it accounts for 2% to 18% of patients hospitalized for pneumonia [1,2]. The overall mortality rate among patients requiring hospitalization for NHAP ranges from 13% to 41% [3,4] and rises to 57% in those with severe NHAP requiring treatment in the intensive care unit [5].

Evaluation of the epidemiology of NHAP has been limited by low bacteriologic yield, poor quality of respiratory specimens, and potential contamination of the upper respiratory tract [6,7]. Many nursing home residents have extensive co-morbidities, which increase their risk for colonization with *Staphylococcus aureus *or enteric Gram-negative rods. However, there is no consensus regarding the best diagnostic strategy in these patients. Clinical features of NHAP are unreliable in establishing accurately the diagnosis of pneumonia in such patients. Quantitative cultures of protected specimen brush (PSB) and bronchoalveolar lavage (BAL) specimens have been used to diagnose severe NHAP [5,8]. However, use of these techniques has been limited by the fact that they are invasive, time consuming, and require fiberoptic bronchoscopy to be available 24 hours per day. Use of quantitative endotracheal aspirates (QEAs) in intubated patients could be viewed as a simple, fast, and cheap way to identify the responsible micro-organisms and, combined with knowledge of local epidemiologic characteristics, can promote early use of appropriate antibiotic therapy. However, the accuracy of QEA in this particular setting has never been assessed. All comparisons of QEAs with other techniques were conducted in patients with ventilator-associated pneumonia [9-11]. The aim of the present study was to compare the diagnostic accuracy of post-intubation QEA with those of PSB and BAL in patients requiring mechanical ventilation for suspected NHAP.

## Materials and methods

### Study population

The study was conducted in a University-affiliated tertiary care center and was derived from a prospective database developed over a six year period extending from January 2000 to December 2005. All nursing home residents presenting to the emergency room with a diagnosis of pneumonia requiring mechanical ventilation were eligible for enrollment if a radiographic infiltrate was present that was compatible with pneumonia, together with symptoms suggestive of lower respiratory tract infection, and any two of the following clinical parameters were present: temperature ≤ 38°C or < 36°C; white blood cells ≤ 10,000/mm^3 ^or < 4,000/mm^3^, or > 15% immature neutrophils (bands) regardless of total peripheral white blood cell count; and purulent respiratory secretions. Patients with severe immunosuppression (solid organ transplantation or steroid therapy ≤ 20 mg/day for more than two weeks), witnessed aspiration, or in whom the pneumonia was an expected terminal event resulting from metastatic cancer were excluded. In addition, patients who were receiving antimicrobial therapy within 72 hours before enrollment were also excluded. The study was approved by the institutional review board and, informed consent was obtained from all participants' next of kin before any invasive procedure was performed.

### Data collection

Data collected on intensive care unit admission included age, sex, co-morbid illnesses, Charlson Index [12], clinical symptoms, and chest radiographic pattern and distribution. The most deranged readings of vital signs, laboratory data, and blood gas analysis within the first 24 hours of admission were also recorded.

### Specimen collection

Within two hours of enrollment in the study, all patients underwent the following protocol. Intravenous midazolam was given before the procedures. The fraction of inspired oxygen was set at 90% or more. None of the patients received local anesthetics. A blind endotracheal aspiration sample was obtained first by sterile means using a 22-inch suction catheter and collected in a mucus collector (Specimen trap, Busse, Hauppauge, NY, USA). Subsequently, a bronchoscopic PSB procedure was performed. A PSB sample was retrieved from the area of maximal inflammation and purulence, as suspected based on chest roentgenogram findings. BAL was then performed from the same segment where PSB had been conducted and analyzed according to standard criteria. All retrieved specimens (endotracheal aspirate, BAL fluid, and PSB specimen) were sent to the microbiology laboratory immediately after collection.

### Microbiologic processing

Endotracheal aspirate and BAL samples were mechanically liquefied and homogenized by vortexing for 1 min with glass beads, followed by centrifuging at 3,000 rpm for 10 min. PSB samples were aseptically cut and placed in a sterile tube containing 1 ml of 0.9% saline solution and vortexed for 1 min. All three types of specimens were serially diluted in 0.9% sterile saline solution. The specimens were then plated into the following agar media: 5% sheep blood, chocolate, and McConkey agar. All cultures were incubated at 37°C under aerobic and anaerobic conditions and in a carbon dioxide enriched atmosphere. Plates were evaluated for growth at 24 and 48 hours and discarded after five days. The number of bacteria in the original sample was expressed in colony-forming units (cfu) per milliliter. All micro-organisms were identified using standard laboratory methods. The threshold for positive culture defining pneumonia was 10^3 ^cfu/ml for PSB or 10^4 ^cfu/ml for BAL, or both. The cutoff points analyzed to establish a positive result for QEA were 10^3^, 10^4^, 10^5^, 10^6^, and 10^7 ^cfu/ml.

### Statistical analysis

Descriptive analysis was performed using the NCSS 2000 software (NCSS Statistical Analysis System, Kaysville, UT, USA). Means were compared using the Student's *t*-test when they were normally distributed and the Mann-Whitney test otherwise. Frequencies were compared using a χ^2 ^test or Fisher's exact test when appropriate. Sensitivity, specificity, false-positive rate, false-negative rate, and overall accuracy were calculated using the standard formulae [13]. Quantitative concordance between techniques was assessed with Pearson's correlation coefficient. Graphic representation of diagnostic parameters was performed, plotting true-positive against false-positive percentages to obtain a receiver operator characteristic curve. All tests were two tailed, and statistical significance was determined at the 5% level.

## Results

Seventy-five patients (45 men and 30 women) were included in the study. The general characteristics of the study population are shown in Table 1. All patients were mechanically ventilated at the time of enrollment. Twenty-one patients had received at least one dose of antimicrobial agent before bronchial samplings.

**Table 1:** Clinical characteristics of the study population

Characteristic	Value
Age (years)	72.7 ± 14.9
Sex (male/female)	45/30
Comorbid diseases	
Coronary artery disease	15 (20)
Chronic pulmonary obstructive disease	31 (41)
Diabetes mellitus	11 (15)
Cerebrovascular accident	27 (36)
Charlson Index	2.3 ± 1.4
Clinical presentation	
Fever	59 (79)
Dyspnea	73 (97)
Cough	32 (43)
APACHE II score	27.8 ± 5.7
PaO_2_/FiO_2_	241 ± 56
Radiologic score	4.6 ± 1.8

Seventy-five patients were included. Values are expressed as number, number (%), or mean ± standard error. APACHE, Acute Physiology and Chronic Health Evaluation; FiO_2_, fractional inspired oxygen; PaO_2_, arterial oxygen tension;

According to our diagnostic criteria, pneumonia was documented in 49 (65%) patients. Fifty-six micro-organisms were isolated at concentrations above the diagnostic thresholds by either PSB or BAL (Table 2). Polymicrobial infection was present in three patients. The most frequently isolated bacteria were *Staphylococcus aureus *(*n *= 13), *Streptococcus pneumoniae *(*n *= 7), and *Escherichia coli *(*n *= 7). There was total agreement in positive bacterial cultures between PSB and BAL in 33 cases. Twenty-six paired cultures were sterile, and in 19 of these cases there was exposure to antimicrobial therapy before the invasive procedures were conducted. Discrepancy was noted in 16 cases, which represented 11 BAL-positive/PSB-negative cases and five BAL-negative/PSB-positive cases.

**Table 2:** Results of quantitative bacterial cultures of BAL and PSB

Pathogen	BAL (≤ 10^4^)	PSB (≤ 10^3^)
*Staphylococcus aureus*	12	8
*Streptococcus pneumoniae*	6	4
*Streptococcus *spp.	5	4
*Haemophilus influenzae*	3	2
*Klebsiella *spp.	5	5
*Escherichia coli*	6	6
*Serratia marcescens*	3	3
*Proteus mirabilis*	3	2
*Enterobacter *ssp.	4	4
*Pseudomonas aeruginosa*	2	1

BAL, bronchoalveolar lavage; PSB, protected specimen brush.

**Figure 1:**
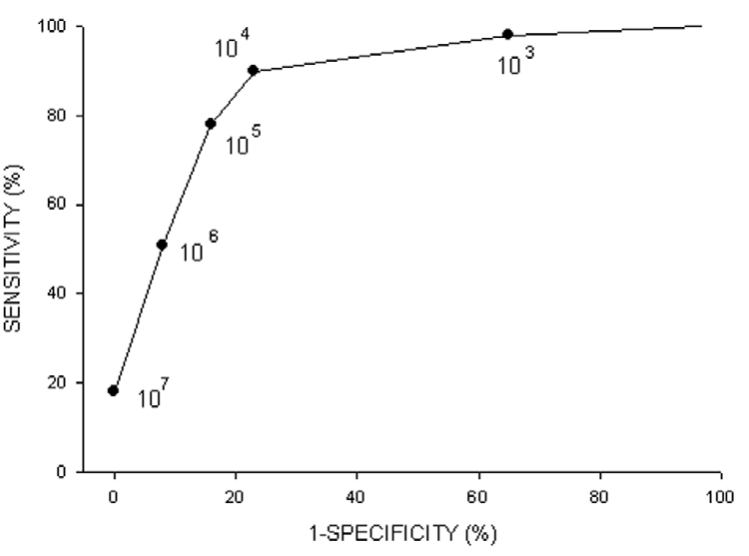
Receiver operator characteristic curve of QEA for the diagnosis of pneumonia. QEA, quantitative endotracheal aspirate.

The operating characteristics of QEA for detecting the presence of pneumonia were obtained over a range of values extending from 10^3 ^through to 10^7 ^cfu/ml (Table 3 and Figure 1). The sensitivity increased from 18% at the threshold of 10^7 ^cfu/ml to 98% at 10^3 ^cfu/ml. Conversely, specificity decreased from 100% at 10^7 ^cfu/ml to 35% at 10^3 ^cfu/ml. The threshold of 10^4 ^cfu/ml yielded the highest accuracy in diagnosis, with a sensitivity of 90% (95% confidence interval [CI] 78% to 97%) and a specificity of 77% (95% CI 56% to 91%). At this threshold, the positive-predictive value was 88% (95% CI 76% to 96%) and the negative predictive value was 80% (95% CI 59% to 93%).

**Table 3:** Operating characteristics of the endotracheal aspirates quantitative cultures

Threshold (cfu/ml)	Sensitivity (%)	Specificity (%)	Accuracy (%)
≤ 10^3^	98 (89–100)	35 (17–56)	76 (65–85)
≤ 10^4^	90 (78–97)	77 (56–91)	85 (78–94)
≤ 10^5^	78 (63–88)	84 (65–96)	80 (69–88)
≤ 10^6^	51 (36–66)	92 (75–99)	65 (54–76)
≤ 10^7^	18 (9–32)	100 (87–100)	47 (35–59)

Values in parentheses are 95% confidence intervals.

**Figure 2:**
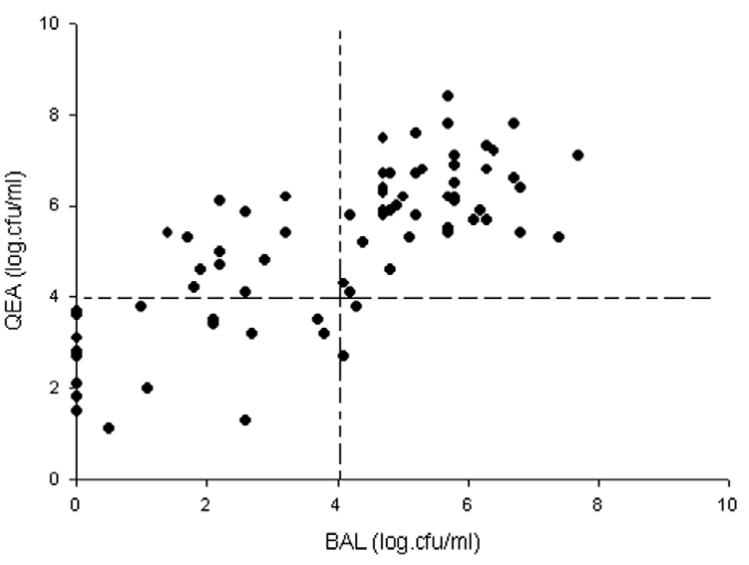
Correlation between quantitative cultures of endotracheal aspirate and BAL specimens. The correlation coefficient (*r*) is 0.77 (*P *< 0.001). BAL, bronchoalveolar lavage; cfu, colony-forming units; QEA, quantitative endotracheal aspirate.

**Figure 3:**
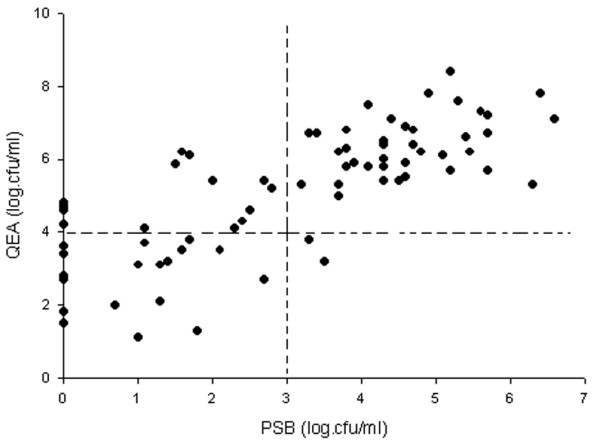
Correlation between quantitative cultures of endotracheal aspirate and PSB specimens. The correlation coefficient (*r*) is 0.71 (*P *< 0.001). PSB, protected specimen brush; cfu, colony-forming units; QEA, quantitative endotracheal aspirate.

Using 10^4 ^cfu/ml as a diagnostic threshold for endotracheal aspirates, QEA coincided with both BAL and PSB in 30 positive culture cases. Partial agreement was present in 14 cases; 10 patients had qualitative concordance between QEA and BAL and four others between QEA and PSB only. In six cases QEA was below the threshold of 10^4 ^cfu/ml for pneumonia documented by BAL or PSB. Comparisons of bacterial counts between endotracheal aspirate and BAL and between endotracheal aspirate and PSB are shown in Figures 2 and 3. The correlation coefficients (*r*) between the quantitative cultures of micro-organisms obtained from endotracheal aspirate and BAL and from endotracheal aspirate and PSB were 0.77 (*P *< 0.001) and 0.71 (*P *< 0.001), respectively.

## Discussion

The results of this study indicate that the best overall accuracy in diagnosing severe NHAP using the endotracheal aspirate culture technique was obtained at a threshold of 10^4 ^cfu/ml. At this cutoff, there was favorable correlation between endotracheal aspirate and PSB cultures and between endotracheal aspirate and BAL cultures. These findings suggest that QEA can be used as a diagnostic tool in severe NHAP where fiberoptic bronchoscopic techniques are not uniformly available or are not practically feasible before the first dose of antimicrobial therapy.

NHAP has traditionally been approached as a disease that resembles community-acquired pneumonia rather than nosocomial pneumonia. The lack of strong epidemiologic data on microbial etiology has resulted in physicians employing diagnostic and treatment strategies in NHAP that are similar to those used in community-acquired pneumonia, and with similar empiric antibiotic coverage. Following the publication of several investigations on the microbial spectrum of pathogens in NHAP [5,14], this approach has shifted drastically toward classifying NHAP as health care-associated pneumonia. Because of the substantial mortality associated with both inadequate and delayed therapy [5,15,16], the most recent American Thoracic Society guideline [17] advises that the initial empirical antibiotic coverage for patients hospitalized with NHAP should have adequate activity against multidrug resistant pathogens. However, indiscriminate use of broad-spectrum antibiotics has been implicated in the emergence of resistant strains in the intensive care unit [18,19]. In response to this, a consensus has emerged emphasizing the implementation of de-escalation therapy or discontinuation of antibiotics once culture results and susceptibility findings are available. Hitherto, the concept of de-escalation has not been tested in patients with severe NHAP, in part because of lack of studies to determine appropriate diagnostic strategies in this segment of the population.

This is the first study to our knowledge to assess the accuracy of QEA, as compared with those of PSB and BAL, in the diagnosis of severe NHAP. Previous studies established that cultures of tracheal aspirates in patients requiring mechanical ventilation for longer than 48 hours may not be accurate [20]. Although negative cultures may have high predictive value, the rate of false-positive results can be high, leading to over-diagnosis of pneumonia and misdiagnosis of etiology. Similarly, quantitative cultures of tracheal aspirates have also yielded mixed results. In intubated patients suspected with nosocomial pneumonia, a colony count ≤ 10^4 ^cfu/ml was found in 80% of patients with clinical pneumonia but also in 61% of patients without obvious pneumonia [11]. These findings stem from the fact that endotracheal tube colonization and biofilm formation occurs in many patients undergoing mechanical ventilation from an early stage. The absence of such a risk factor would minimize the risk for tracheal aspirate contamination, resulting in improved diagnostic accuracy. Indeed, at a threshold of 10^4 ^cfu/ml, we were able to demonstrate good sensitivity (90%), specificity (77%), and negative predictive value (80%) for QEA as compared with BAL and PSB. It is noteworthy that one-third of our patients had received antibiotics before samplings, which could be regarded as a potential bias because it could have led to a high rate of false-negative results. Lowering the threshold value has been proposed as an alternative for patients receiving antibiotic therapy in patients suspected of having ventilator-associated pneumonia [21]. In this study we were unable to confirm this approach, in part because of the lack of histopathologic specimens.

One of the major advantages of endotracheal cultures is that they exclude certain types of infection when the organism is absent [22]. For example, absence of *Pseudomonas *in an endotracheal aspirate makes it unlikely that this organism is the cause of an infection. Conversely, it could be argued that the lack of micro-organisms on Gram staining in nursing home patients suspected of having NHAP might suggest the presence of atypical pathogens. Although outbreaks of pneumonia caused by atypical pathogens can occur among nursing home residents, the incidence of these infections is relative low in this population [5,23]. Nonetheless, appropriate testing should be performed and empiric coverage should be provided when it is highly suspected.

This study has a number of strengths, including use of each patient as his or her own control and using explicitly defined criteria for quantitative culture positivity. However, it has a number of limitations, as do all studies in this discipline. We relied upon bronchoscopic techniques (PSB and BAL) as gold standards, but these methods are not 100% sensitive or specific. It has been suggested that only the combined results of histologic examination and quantitative cultures of lung tissue are sufficiently powerful to rule in or rule out the presence of pneumonia. Clearly, such a highly invasive sampling approach would not be practical in a frail population. Without an indisputable and easily obtainable reference, calculations of sensitivity and specificity will remain problematic. Finally, the impact of QEA findings on antibiotic therapy and patient outcomes was not assessed. Pending further validation, we thought it would be too premature to make any antibiotic recommendation for the management of severe NHAP based on the present study alone.

## Conclusion

The present study shows that QEA using a cutoff point of 10^4 ^cfu/ml represents a practical diagnostic method in patients with severe NHAP. This modality may be useful as a first line technique for intubated NHAP patient with clinical suspicion of pneumonia when it is more convenient to perform prior to administration of antibiotic therapy.

## Key messages

• Accurate diagnosis of severe NHAP remains a challenge for clinicians in intensive care unit settings.

• Quantitative cultures of samples obtained by BAL and PSB are considered to be the tests that offer the best diagnostic accuracy, but these methods are invasive, expensive, and not uniformly available.

• Quantitative endotracheal aspirates in cases of severe NHAP correlate well with quantitative bronchoscopic PSB and BAL in intubated nursing home patients who are suspected of having clinical pneumonia.

• QEA may be a reliable alternative to PSB or BAL in the setting of severe NHAP.

## Abbreviations

BAL = bronchoalveolar lavage; cfu = colony-forming units; CI, confidence interval; NHAP = nursing home-acquired pneumonia, PSB = protected specimen brush; QEA = quantitative endotracheal aspirate.

## Competing interests

The authors declare that they have no competing interests.

## Authors' contributions

AES conceived of the study, collected all respiratory samples, and edited the manuscript. MEA participated in data collection and data analysis. LAP assisted with the acquisition of the respiratory samples, performed quality assurance, and provided a first draft of the manuscript. CRM recruited eligible patients, and collected clinical and microbiologic data.
